# Effect of *Moringa oleifera* on hematological parameters of calves reared in industrial fluorotic area

**DOI:** 10.14202/vetworld.2015.1364-1369

**Published:** 2015-11-28

**Authors:** Kruti Debnath Mandal, M. R. Das, M. Pati, P. D. Pati, A. R. Gupta, R. C. Patra, S. K. Senapati

**Affiliations:** 1 Department of Clinical Medicine, Ethics and Jurisprudence, College of Veterinary Science and Animal Husbandry, Orissa University of Agriculture and Technology, Bhubaneswar - 751 003, Odisha, India; 2 Department of Poultry Science, College of Veterinary Science and Animal Husbandry, Orissa University of Agriculture and Technology, Bhubaneswar - 751 003, Odisha, India

**Keywords:** aluminum smelter, boron, calcium, calves, fluorosis, *Moringa oleifera*

## Abstract

**Aim:**

The present study was undertaken to evaluate the ameliorative potential of dried *Moringa oleifera* fruit powder in fluorosis affected calves reared around the vicinity of aluminium smelter plant.

**Materials and Methods:**

Total 107 calves were screened on the basis of clinical signs and higher plasma fluoride (more than 0.2 ppm) level for evidence of fluorosis. Out of that, 90 samples found positive and from them 18 calves of 6-12 months age group were selected and divided equally into three groups named as Group II, III, and IV. Group II remained as disease control group whereas Group III calves were supplemented with dried *M. oleifera* fruit powder of 25 g/calve for 60 days. Group IV calves were supplemented with calcium carbonate at 100 mg/kg body weight and boric acid at 10 mg/kg for the same experimental period. Group I consisted of six numbers of healthy calves taken from the non-fluorotic zone, i.e. Bhubaneswar. Plasma fluoride level, hemoglobin (Hb), packed cell volume (PCV), total leukocyte count (TLC), differential count (DC), total erythrocyte count, mean corpuscular volume (MCV), mean corpuscular Hb (MCH), and MCH concentration (MCHC) were estimated on day 0, 30, and 60 of the experiment.

**Results:**

Supplementation of dried *M. oleifera* fruit powder to fluorosis affected calves resulted in significant reduction in plasma fluoride level and increase in Hb%, PCV, TLC and altered DC. Similar results were also recorded in calcium+boron group, except PCV and Hb. No significant changes were observed in MCV, MCH, and MCHC values.

**Conclusion:**

The present study concluded that supplementation of dried *M. oleifera* fruit powder daily for 60 days has shown protection against chronic fluoride toxicity in calves.

## Introduction

Fluoride acts as a double edged knife. At low concentration (<1 ppm), fluoride helps to prevent the formation of dental carries but cumulative ingestion of small but toxic amount results in dental as well as skeletal lesions, commonly called as fluorosis [1]. During last decades, numerous reports of fluorosis in animals and humans from India [2], China [3], USA, Australia [4], and other countries were observed. In India, common sources of fluoride intoxications are water, fodder, fluoride rich effluents, dust and smoke from aluminium smelters plants, copper, glass, iron, super phosphate fertilizers plants and brick kilns areas [5,6].

The maximum susceptibility to fluoride toxicosis was found in bovines followed by equines, flocks (goats and sheep) and Camelids [7,8]. Among them, calves were considered as bioindicator for fluoridated water and more susceptible to dental than skeletal fluorosis [8,9].

Several treatment methods have been tried to wade off the fluoride level including inorganic, organic and techniques like reverse osmosis, nanofiltration, electrodialysis, donanan dialysis, ultrafiltration, ion exchange, and adsorption [10,11]. Ethno-veterinary medical substances such as fruits of amla (*Emblica officinalis*) [12], fruit pulp of tamarind (*Tamarindus indica*) [13], *Primula heterochroma* Stapf extracts [14], black tea [15], Jambul fruit pulp extract [16], nettle extracts [17] have beneficial effect over fluoride intoxication. Further, Ranjan *et al*. [18] found the beneficial effect of aqueous extract of *Moringa* seeds and tamarind pulps to mitigate fluoride toxicity in rabbit.

Hence, the present study assessed the ameliorative efficacy of dried fruit powder of *Moringa oleifera* in calves reared around aluminium smelter plant in Odisha, India by estimating alterations in plasma fluoride level and hematological parameters as biomarkers before and after supplementation.

## Materials and Methods

### Ethical approval

The experimental procedures have been conducted in accordance with the guidelines laid down by the Institutional Ethics Committee.

### Study site

The study site was selected on the basis of the previous report of endemic fluorosis in the villages around close proximity of aluminium smelter plant in Odisha, India. The present study site is located 133 km away from Bhubaneswar city at latitude 20.83°N and longitude 85.15°.

### Plant materials

Semi ripen fruits of *M. oleifera* were procured from local market. These fruits were washed with clean water to remove dirt and soil. The fruits having any external visible lesions or decomposed ones were discarded. The tip of both ends of fruits were cut and removed. The fruits were now cut into 3-5 cm pieces and dried at 60°C up to a constant mass. These dried materials further processed into powder form by passing through feed grinder and kept in air tight packets for further use. The 100 g of *M. oleifera* fruit yielded 6-8 g of powder.

### Animals and experimental design

Total 107 calves were screened on the basis of clinical signs and higher plasma fluoride (more than 0.2 ppm) level for evidence of fluorosis. Out of that, 90 samples found positive and from them 18 calves of 6-12 months age group were selected and divided equally into three groups (six animals each) named as Group II, III, and IV respectively. Body weight (b.wt.) of each of these selected animals was nearly equal to 100 kg. Six healthy calves residing in fluorosis free zones (Bhubaneswar) were selected and taken as a healthy group (Group I). Group II animals received no treatment and were selected as disease control. Group III animals were supplemented orally, daily with *M. oleifera* fruit powder at 250 mg/kg b.wt. Group IV calve were treated with calcium along with boron at 100 mg/kg b.wt. and 10 mg/kg b.wt., respectively. The duration of experimentation was of 60 days.

### Sample collection

Blood samples were collected from a jugular vein of both affected and healthy cattle during the early morning on day 0, 30, and 60 of the experiment. About 5 ml of blood was collected from each selected animal for hematological estimation. Plasma was separated from the heparinized blood samples for the estimation of fluoride.

### Evaluation of level of fluoride and hematological parameters

The fluoride concentration of plasma samples were measured by ion specific potentiometry, using total ionic strength adjustment buffer and following the method adopted by Cernik *et al*. [19] with modifications using a portable fluoride ion-specific electrode (Orion Model 96-09) and ISE meter (Orion Model-290A). The detection range of the instrument is in between 0.019 and 1900 ppm. Calibration of the instrument was made using five freshly prepared working standards. The accuracy and precision of the measurements were maintained by repeated analysis of the reference standard procured from Orion Research Incorporated Laboratory, USA.

After collecting the heparinized blood samples from all fluorotic calves, hemoglobin (Hb) (g/dL), total erythrocyte count (TEC) and total leukocyte count (TLC) were estimated as stated by Benjamin [20]. Packed cell volume (PCV) was determined using Wintrobe’s method as described by Coles [21]. Differential leukocyte count (DLC) was made on blood films stained by Giemsa staining method [22]. Erythrocyte indices like mean corpuscular volume (MCV), mean corpuscular Hb (MCH), MCH concentration (MCHC) were calculated as described by Schalm *et al*. [22].

### Statistical analysis

The data obtained from each parameter were compiled and statistically analyzed to find out the mean, standard error, and significant difference of mean values within the group and between the groups at p≤0.05 as per standard method described by Snedecor and Cochran [23] and SPSS software version 22.

## Results and Discussions

The chronic fluoride poisoning can be prevented by two means, i.e., either by reducing ingestion or through increasing elimination/excretion of fluoride from the body. Various chelating agents have been tried to increase fluoride excretion from body. In this study, we have evaluated the effect of *M. oleifera* on hematological parameters of fluoride affected calves reared in vicinity of aluminum smelter plant. *M. oleifera* fruit not only have chelating property but also have other constituents that protect oxidative damage to the hematopoietic system as well reduce cytotoxic effect of fluoride intoxication.

Supplementation of *M. oleifera* fruit powder was able to reduce the plasma fluoride level in affected calves. Interference with fluoride absorption from the gut might have played a role in reducing plasma fluoride concentrations. The lower molecular weight water soluble proteins in *Moringa* seeds have a strong positive charge that attracts highly electronegative fluoride ions resulting in formation of flocculants [24]. Furthermore, the presence of tannins, fibers and high concentration of minerals in *Moringa* like calcium, aluminum, phosphorus, manganese, potassium, copper, and iron [25] which are reported to form insoluble complexes with fluoride in the gut.

Fluoride affected calves revealed significantly (p<0.05) higher level of plasma fluoride as compared to normal calves at different observation periods. Significant (p<0.05) reduction in plasma fluoride concentration was recorded after supplementation of *M. oleifera* in the diet of fluoride affected calves. Calcium and boron treated group revealed a significant decrease in concentration of fluoride as compared to non-treated fluorotic calves.

Hematological parameters of calves of different treatment groups are presented in Table-1. Significant lowered Hb content, PCV and TLC were observed in fluorotic calves as compared to healthy calves. Supplementation of *M. oleifera* and calcium with boron significantly increased the level of Hb, PCV, and TLC in fluorotic calves as compared to the non-treated group at the end of the experiment.

**Table-1:** Plasma fluoride and hematological parameters in different groups of calves.

Group (n=10)	Day 0	Day 30	Day 60
Plasma fluoride concentration (ppm)			
I	0.084±0.004^A^	0.086±0.007^A^	0.087±0.011^A^
II	0.523±0.009^B^	0.537±0.009^C^	0.542±0.012^C^
III	0.531±0.025^cB^	0.277±0.027^bB^	0.221±0.027^aB^
IV	0.549±0.029^cB^	0.295±0.020^bB^	0.215±0.019^aB^
Hb (g/dL)			
I	10.933±0.341^B^	11.100±0.333^B^	11.100±0.276^C^
II	7.566±0.370^A^	7.270±0.400^A^	7.368±0.371^A^
III	7.333±0.312^A^	7.516±0.258^A^	8.695±0.386^Bb^
IV	7.333±0.363^A^	7.866±0.256^A^	8.246±0.310^AB^
PCV (%)			
I	32.833±1.108^B^	33.000±1.154^B^	33.500±0.991^B^
II	22.666±1.256^A^	23.333±1.308^A^	22. 833±1.137^A^
III	22.833±1.470^A^	23.333±1.382^A^	27.166±1.740^A^
IV	22.666±1.584^A^	24.000±1.527^A^	25.000±1.211^A^
TLC (10^3^per cu mm of blood)			
I	8933.333±294.863^B^	8475.00±243.84^B^	8483.333±209.629^C^
II	5575.000±224.629^A^	5375.000±106.262^A^	5933.000±392.994^A^
III	5533.333±463.621^aA^	5700.000±157.056^aA^	7550.000±232.02^bB^
IV	5483.333±149.257^aA^	5783.333±177.795^aA^	6850.000±172.723^bB^

Group I: Healthy control; Group II: Disease control; Group III: *Moringa* treatment; Group IV: Ca+B treatment. Values (mean±SE) having no common superscript (small letter in a row and capital letter in a column) differ significantly at p<0.05. SE=Standard error, Ca+B=Calcium+boron, TLC=Total leukocyte count, Hb=Hemoglobin, PCV=Packed cell volume

DLCs of different experiment groups are presented in Table-2. Significant increase in lymphocyte and eosinophil count was observed in fluorotic calves in comparison to normal calves. Whereas neutrophil count was significantly lower in fluorotic cows as compared to healthy calves. Supplementation of *Moringa* (Group III) and calcium+boron (Ca+B) (Group IV) significantly reduces the lymphocyte and eosinophil count and increase neutrophil count in affected calves at different observation periods of the experiment as compared to calves received no treatment.

**Table-2:** Differential leukocyte counts in different groups of calves.

Group (n=10)	Day 0	Day 30	Day 60
Lymphocyte count (%)			
I	57.833±0.401^A^	58.166±0.749^A^	58.166±0.401^A^
II	66.500±1.118^B^	66.330±1.145^B^	66.166±1.376^C^
III	67.670±1.837^bB^	63.670±0.76^aB^	62.340±0.557^aB^
IV	68.170±1.351^bB^	64.170±0.909^aB^	61.840±0.401^aB^
Neutrophil count (%)			
I	38.833±0.166^B^	37.500±0.718^C^	37.166±0.477^C^
II	22.830±1.013^A^	23.833±0.833^A^	22.000±1.549^A^
III	23.666±0.421^aA^	26.833±0.792^bB^	31.340±0.802^cB^
IV	23.000±0.447^aA^	26.833±1.137^bB^	31.170±0.477^cB^
Eosinophils count (%)			
I	2.161±0.307^A^	3.000±0.258^A^	2.167±0.360^A^
II	9.833±0.703^B^	8.833±0.477^B^	9.666±0.421^C^
III	9.333±0.614^bB^	7.833±0.401^bB^	4.833±0.542^aB^
IV	8.5±1.087^bB^	7.833±0.307^bB^	4.500±0.342^aB^

Group I: Healthy control; Group II: Disease control; Group III: *Moringa* treatment; Group IV: Ca+B treatment. Values (mean±SE) having no common superscript (small letter in a row and capital letter in a column) differ significantly at p<0.05. SE=Standard error, Ca+B=Calcium+boron

There was significant reduction in Hb, TLC and PCV in fluorotic calves than healthy ones. The presence of anemia in fluoride affected animal was also reported in calves [26,27], buffaloes, cows [28], and goats [29]. Decrease in TLC was also observed by Swarup and Singh [28] in cattle, in goats [29] and in mice [30]. It is known that fluoride intoxication depresses bone marrow activity in cattle [31] resulting in anemia due to reduced erythropoiesis. Fluoride-induced disorders in hematopoietic organs in mice and in human hematopoietic progenitor cells are on record [32]. Decrease in Hb may be also possibly, due to toxic effect of fluoride on the serum level of iron and poor retention of iron [33]. Significant PCV changes in the study might be due to toxic effects of fluoride on the red blood cells (RBC) cell membrane and subsequently shrinkage of cell. Studies on cattle, goats and sheep in relation to hematological alterations in fluorotic animals by various authors also revealed similar changes [29,33].

In the present investigation, there was increase in Hb, TLC and PCV value in both the treatment groups after 60 days of treatment. This might be due to prevention of oxidative damage to cell membrane of RBC by boron [26] in Ca+B supplementation group. However, the more significant increase in *M. oleifera* supplementation group than Ca+B might be due to high Fe, Cu and antioxidant like ascorbic acids, polyphenols, flavonoids, and organosulfur compounds present in *Moringa* [34].

A significant increase in lymphocyte %, eosinophil %, and decrease in neutrophil % was revealed in the study which was also reported by other workers [35]. This may be due to constant ingestion of toxic dose of fluorine over the period of experiment. But significant alterations in above parameters toward their normal values were observed in both the treatment groups. This alteration may be attributed by increased fluoride excretion from body system and cytoprotective effect of *M. oleifera* fruit powder.

Erythrocyte indices like TEC, MCV, MCH, and MCHC were estimated to know the type of anemia produced due to fluoride intoxication. MCV classifies the anemia as macrocytic, normocytic and microcytic based on erythrocyte volume. Similarly, MCH expresses the weight of Hb in single RBC cell whereas MCHC dictates concentration of Hb in PCV. Both MCH and MCHC values help to designate anemia as hypochoromic, normochromic, or hyperchromic type. Low level of TEC (Table-3) established the occurrence of anemia in affected calves. However, no significant differences in erythrocyte indices, like MCV, MCH, and MCHC values, were suggestive of occurrence of a normochromic normocytic type of anemia in those calves. The result of our study is in accord with the results of Gill and Dumka [27].

**Table-3:** Different erythrocyte indices in different groups of calves.

Group (n=10)	Day 0	Day 30	Day 60
TEC (10^6^ per cu mm of blood)			
I	7.530±0.349^B^	7.553±0.408^B^	7.673±0.481^C^
II	4.811±0.275^A^	5.053±0.285^A^	4.843±0.198^A^
III	4.818±0.218^Aa^	5.335±0.244^Aab^	5.791±0.280^Bb^
IV	4.920±0.133^A^	5.351±0.205^A^	5.600±0.251^AB^
MCV (fL)			
I	43.933±1.998	44.092±2.013	44.318±2.389
II	47.406±2.382	46.321±1.811	47.180±1.669
III	47.559±3.152	43.606±0.834	47.011±2.673
IV	46.128±3.075	44.799±2.137	44.629±0.679
MCH (pg/dL)			
I	14.665±0.764	14.888±0.832	14.684±0.749
II	15.772±0.283	14.450±0.622	15.219±0.483
III	15.332±0.842	14.138±0.287	15.085±0.647
IV	14.918±0.663	14.741±0.405	14.796±0.508
MCHC (g/dL)			
I	33.341±0.565	33.702±0.675	33.163±0.349
II	33.546±1.173	31.236±0.878	32.358±1.020
III	32.470±1.282	32.504±1.063	32.360±1.347
IV	32.648±0.993	33.162±1.260	33.166±1.105

Group I: Healthy control; Group II: Disease control; Group III: *Moringa* treatment; Group IV: Ca+B treatment. Values (mean±SE) having no common superscript (small letter in a row and capital letter in a column) differ significantly at p<0.05. SE=Standard error, Ca+B=Calcium+boron, TEC=Total erythrocyte count, MCV=Mean corpuscular volume, MCH=Mean corpuscular hemoglobin, MCHC=Mean corpuscular hemoglobin concentration

## Conclusions

This present study reveals that dried fruit powder of *M. oleifera* is quiet effective in reducing the plasma fluoride content, correcting anemia and improving immune status of calves. Thus, present study gives a bird’s eye view of usage of local and cheap plant products like *M. oleifera* dry fruit powder in alleviating hazardous effect of fluorosis. However, further studies needs to be done in more and more fluoride affected animals to validate this study and also to evaluate the presence of active principles in this plant product responsible for ameliorating effect.

## Authors’ Contributions

The present study is a thesis part of M.V.Sc. degree of KDM. MRD and RCP planned the study and KDM done the research under the guidance of ARG and MRD. ARG and SKS guided in statistical analysis. MP and PDP helped in data collection and estimation process of this experimentation. All authors participated in draft and revision of the manuscript. All authors read and approved the final manuscript.

## References

[1] Warren J.J., Levy S.M., Broffitt B., Cavanaugh J.E., Kanellis M.J., Weber-Gasparoni K.J. (2009). Considerations on optimal fluoride intake using dental fluorosis and dental caries outcomes –A longitudinal study. Public Health Dent.

[2] Arif M., Hussain J., Hussain I., Kumar S. (2013). An investigation of fluoride distribution in ladnu block of Nagaur district, Central Rajasthan. World Appl. Sci. J.

[3] Li F.C., Guanb Z.Z. (2014). Synergistic intoxication with aluminum and fluoride in patients in an area of coal burning endemic fluorosis. Fluoride.

[4] Jubb T.F., Annand T.E., Main D.C., Murphy G.M. (1993). Phosphorus supplementation and fluorosis in cattle - A Northern Australian experience. Aust. Vet. J.

[5] Patra R.C., Dwivedi S.K., Bhardwaj B., Swarup D. (2000). Industrial fluorosis in cattle and buffalo around Udaipur, India. Sci. Total Environ.

[6] Gupta A.R., Dey S., Swarup D., Saini M., Saxena A., Dan A. (2013). Ameliorative effect of *Tamarindus indica* L. on biochemical parameters of serum and urine in cattle from fluoride endemic area. Vet. Arch.

[7] Han-Bo Shi Y., Han B., Shi Y. (2001). Studies on the alleviation of bovine endemic fluorosis. J. Yangzhou Univ. Nat. Sci.

[8] Choubisa S.L. (2014). Bovine calves as ideal bio-indicators for fluoridated drinking water and endemic osteo-dental fluorosis. Environ. Monit. Assess.

[9] Shupe J.L., Christofferson P.V., Olson A.E., Allred E.S., Hurst R.L. (1987). Relationship of cheek tooth abrasion to fluoride-induced permanent incisor lesions in livestock. Am. J. Vet. Res.

[10] Jorfi S., Kalantary R.R., Bandpi A.M., Jaafarzadeh N., Esrafili A., Alaei L. (2011). Fluoride removal from water by adsorption using bagasse, modified bagasse and chitosan. Iran. J. Health Environ.

[11] Zazouli M.A., Balarak D., Karimnezhad F., Khosravi F. (2014). Removal of fluoride from aqueous solution by using of adsorption onto modified Lemna minor: Adsorption isotherm and kinetics study. J. Mazand. Univ. Med. Sci.

[12] Rao M.V., Thakura S.B. (2013). Effects of melatonin and amla antioxidants on fluorideinduced genotoxicity in human peripheral blood lymphocyte cells. Fluoride.

[13] Ekambaram P., Namitha T., Bhuvaneswari S., Aruljothi S., Vasanth D., Saravanakumar M. (2010). Therapeutic efficacy of *Tamarindus indica* (l) to protect against fluoride-induced oxidative stress in the liver of female rats. Fluoride.

[14] Alinezhad H., Zare M., Nabavi S.F., Naqinezhad A., Nabavia S.M. (2011). Assessing the protective effect of *Primula heterochroma* stapf extracts against sodium fluoride-induced hemolysis in rat erythrocytes. Fluoride.

[15] Trivedi M.H., Verma R.J., Sangai N.P., Chinoy N.J. (2011). Black tea extract mitigation of naf-induced lipid peroxidation in different regions of mice brain. Fluoride.

[16] Ahmad K.R., Nauroze T., Raees K., Abbas T., Kanwal M.A., Noor S., Jabeena S. (2012). Protective role of jambul (*Syzygium cumini*) fruit-pulp extract against fluoride-induced toxicity in mice testis: A histopathological study. Fluoride.

[17] Gutowska I., Jakubczyk K., Dec K., Baranowska-Bosiacka I., Drozd A., Janda K., Wolska J., Łukomska A., Dębia K., Chlubekb D. (2014). Fruit cluster on the synthesis of pro-inflammatory agents in hepatocytes treated with fluoride. Fluoride.

[18] Ranjan R., Swarup D., Patra R.C., Chandra V. (2009). *Tamaridus indica* L. *Moringa oleifera* extract administration ameliorates fluoride toxicity in rabbits. Indian J. Exp. Biol.

[19] Cernik A.A., Cooke J.A., Hall R.J. (1970). Specific ion electrode in the determination of urinary fluoride. Nature.

[20] Benjamin M.H. (1978). Outline of Veterinary Clinical Pathology.

[21] Coles E.H. (1986). Veterinary Clinical Pathology.

[22] Schalm O.W., Jain N.C., Caroll E.J. (1986). Veterinary Hematology.

[23] Snedecor G.W., Cochran W.G. (1989). Statistical Methods.

[24] Mangale S.M., Chonde S.G., Rout P.D. (2012). Use of *Moringa oleifera* (Drumstick) seed as natural absorbent and an antimicrobial agent for ground water treatment. Res. J. Recent Sci.

[25] Anjorin T.S., Ikokoh P., Okolo S. (2010). Mineral composition of *Moringa oleifera* leaves, pods and seeds from two regions in Abuja, Nigeria. Int. J. Agric. Biol.

[26] Bharti V.K., Gupta M., Lall D., Kapoor V. (2007). Effects of boron on haemogram and urine profile in buffalo calves fed a high F ration. Fluoride.

[27] Gill K.K., Dumka V.K. (2013). Haematological alteration induced by subchronic oral exposure of buffalo calves to fipronil and fluoride. Fluoride.

[28] Swarup D., Singh Y.P. (1989). Bovine fluorosis in a brick kiln congested zone. Indian J. Vet. Med.

[29] Vinay K., Verma P.K., Pankaj N.K., Kumar J., Raina A.R., Srivastava A.K. (2009). Haematological profile of subacute oral toxicity of fluoride and ameliorative efficacy of aluminium sulphate in goats. Toxicol Int.

[30] Rao V.B., Vidyunmala S. (2010). Cumulative Effect of fluoride on hematological indices of mice *Mus norvegicus albinus*. Am. Eur. J. Toxicol. Sci.

[31] Radostitis O.M., Gay C.C., Blood D.C., Hinchcliff K.W. (2000). Veterinary Medicine.

[32] Machalinska A., Wiszniewska B., Tarasiuk J., Machalinski D. (2002). Morphological effect of sodium fluoride on hematopoetic organs in mice. Fluoride.

[33] Hoogstratten B., Leone N.C., Shupe J.L., Greenwood D.A., Lieberman L. (1965). Effect of fluorides on hemopoietic system, liver and thyroid gland in cattle. J. Am. Med. Assoc.

[34] Aja P.M., Nwachukwu N., Ibiam A.M., Igwenyi I.O., Onu P.N. (2014). Comparative evaluation of transaminases and alkaline phosphatase activities in Albino rats administered aqueous, ethanolic and methanolic extracts of *Moringa oleifera* seeds locally grown in Abakaliki, Nigeria. J. Biol. Chem. Res.

[35] Agha F.E., El-Badry M.O., Hassan D.A.A., Elraouf A.A. (2012). Role of vitamin E in combination with methionine and L-carnosine against sodium fluoride-induced hematological, biochemical, DNA damage, histological and immuno-histochemical changes in pancreas of albino rats. Life Sci. J.

